# GPR139, an Ancient Receptor and an Emerging Target for Neuropsychiatric and Behavioral Disorders

**DOI:** 10.1007/s12035-025-04828-2

**Published:** 2025-03-18

**Authors:** Minyu Chan, Satoshi Ogawa

**Affiliations:** Present Address: Jeffrey Cheah School of Medicine and Health Sciences, Monash University Malaysia, Jalan Lagoon Selatan, Bandar Sunway, 47500 Subang Jaya, Selangor Malaysia

**Keywords:** GPR139, GPCR physiology, Habenula, Neuropsychiatric disorders

## Abstract

GPR139 is an orphan G-protein-coupled receptor that is predominantly expressed in several midbrain regions, e.g., the habenula, striatum, and hypothalamus. *GPR139* gene is highly conserved across vertebrate phylogenetic taxa, suggesting its fundamental importance in neurophysiology. Evidence from both animal studies and human genetic association studies has demonstrated that dysregulation of GPR139 expression and function is linked to aberrant behaviors, cognitive deficits, alterations in sleep and alertness, and substance abuse and withdrawal. Animal knockout models suggest that GPR139 plays an anti-opioid role by modulating the signaling activity of the μ-opioid receptor (MOR), as well as the intensity of withdrawal symptoms and nociception in behavioral paradigms. Modulation of GPR139 activity by surrogate agonists such as TAK-041 and JNJ-63533054 has shown promising results in experimental models; however, the use of TAK-041 in clinical trials has produced heterogeneous effects and has not met the intended primary endpoint. Here, we highlight current in vitro and in vivo studies of GPR139, its potential physiological roles, and therapeutic potential in the pathophysiology of neuropsychiatric and behavioral disorders. This review aims to focus on the current knowledge gaps to facilitate future studies that will contribute to the understanding of GPR139 as a therapeutic target for neuropsychiatric and behavioral disorders.

## GPR139: Current Consensus

G-protein-coupled receptors (GPCRs) represent the largest and most diverse yet structurally similar group of receptors present in eukaryotes, i.e., they possess seven transmembrane helices with an extracellular N-terminus and an intracellular C-terminus; hence, GPCRs are also known as 7-transmembrane receptors (7-TMs) [1]. Owing to their functional diversity, there are several known classifications of GPCRs: (i) sequence similarity family-based classification, (ii) ligand-based functionality classification, and (iii) endogenous ligand-based classification [2]. However, there are still more than 100 GPCRs whose endogenous ligands have not yet been identified; these GPCRs are known as orphan GPCRs. Simply put, orphan receptors are proteins activated by unidentified or unclassified ligands with unknown physiological functions; however, they are structurally similar to known receptors. GPR139 has been classified as an orphan receptor for the past 20 years.

*GPR139* is located on chromosome 16 in humans, and it was first identified by Takeda et al. via human genome sequence analysis in 2002 [3]. Its exclusive expression in brain tissue was first reported in 2005, where it was then termed *GPRg1* [4]. Previous studies reported its predominant expression in midbrain regions of multiple vertebrate models, i.e., the highest expression is in the habenula, followed by the striatum, and the lowest expression is in the medulla oblongata and ventral tegmental area (VTA), which will be further elaborated in the “GPR139 in Animal Studies” section [4–10]. Its remarkable conservation across vertebrate taxa suggests its fundamental importance in neurophysiology; thus, it has gained traction as a therapeutic target for neuropsychiatric disorders, but its physiological role remains elusive.

Here, we review the current knowledge on GPR139 and explore the potential physiological roles of GPR139 in the context of neuropsychiatric and behavioral disorders. This review aims to facilitate future studies in contextual analyses and experimental designs, contributing to the understanding of GPR139 as a therapeutic target for neuropsychiatric and behavioral disorders.

### Human Gene‒Phenotype Studies

Genome-wide association studies (GWASs) and transcriptomic profiling analyses are useful for examining the gene‒phenotype relationship in neuropsychiatric and behavioral disorders, where a genetic variant can be associated with a trait or phenotype in a clinical context. *GPR139* has been associated with a range of specific phenotypes observed across major depressive disorder (MDD), schizophrenia, and attention-deficit/hyperactivity disorder (ADHD), including insomnia, lateral ventricle volume changes, and alcohol abuse (refer to Table 1 for the included EBI GWAS accession IDs), suggesting that GPR139 is a potential therapeutic target for these neuropsychiatric disorders and their comorbidities. Multiple SNPs have been reported at the *GPR139* loci for insomnia [11–13]. *GPR139* has been significantly associated with the number of nocturnal sleep episodes across two independent studies [12, 14]. Ebejer et al. identified *GPR139* as a gene associated with inattention in juvenile ADHD [15]. Brouwer et al. identified *GPR139* as a locus of interest associated with age-independent changes in lateral ventricle volume in a European cohort, a prominent trait of schizophrenia and MDD [16]. Dunn et al. reported a potential association between *GPR139* and depressive symptoms in a cohort of African American women [17]. *GPR139* risk variants were also found to be associated with alcohol use disorder in two independent studies [18, 19]. In transcriptomic studies, an SNP array conducted by Castellani et al. reported *GPR139* as a candidate gene for schizophrenia in monozygotic twins [20]. Interestingly, an in silico analysis performed by Kaushik et al. reported either an up- or downregulation of *GPR139* expression in a number of cancers, including breast cancer, glioblastoma, pheochromocytoma, and paraganglioma [21]. However, there was no information on neuropsychiatric comorbidities in the study by Kaushik et al.

**Table 1:** *GPR139* risk alleles from GWAS associated with traits directly correlated with MDD, schizophrenia, and ADHD

Trait/phenotype	Risk allele in *GPR139*	*p* value	Citations	EBI GWAS Accession ID (if available)
**Insomnia**	rs1011939	3e^−12^	Watanabe et al., 2022 [11]	GCST90131901
	rs11074422	4e^−11^		
	rs114285994	7e^−9^		
	rs11643465	3e^−11^		
	rs12102869	3e^−14^		
	rs1925493	1e^−14^		
	rs55963159	3e^−13^		
	rs56120127	1e^−14^		
	rs57790054	1e^−8^		
	rs72772728	8e^−10^		
	rs72772751	4e^−8^		
	rs8046312	8e^−12^		
	rs8054082	8e^−20^		
	rs1886715	1e^−8^	Schoeler et al., 2023 [13]	GCST90267286
	rs67501351	5e^−11^	Jansen et al., 2019 [12]	GCST007988
**Number of nocturnal sleep episodes**	rs28651105	5e^−8^	Jansen et al., 2019 [12]	GCST007982
	rs8045740	1e^−3^	Jones et al., 2019 [14]	N/A
**Inattention**	rs10521114	8e^−6^	Ebejer et al., 2013 [15]	GCST001943
	rs10521115	8e^−6^		
	rs11642377	7e^−6^		
	rs11647507	7e^−6^		
	rs12596252	3e^−6^		
	rs12919130	2e^−6^		
	rs12924103	7e^−6^		
	rs12926725	3e^−6^		
	rs12926729	7e^−6^		
	rs12931939	6e^−6^		
	rs1902813	3e^−6^		
	rs2608200	6e^−6^		
	rs6497436	6e^−6^		
	rs7185264	7e^−6^		
	rs7201408	7e^−6^		
**Lateral ventricle volume change**	rs72772746	7e^−8^	Brouwer et al., 2022 [16]	GCST90128593
**Alcohol use disorder**	rs72771074	5e^−8^	Sanchez-Roige et al., 2019 [18]	GCST006718
	rs2764771	4e^−10^	Liu et al., 2019 [19]	GCST007461
**Obesity**	rs12446632	2e^−10^	Berndt et al., 2013 [118]	GCST001953
	rs11639988	4e^−9^		
	rs12446554	4e^−12^		
	rs8046312	3e^−6^	Warner et al., 2021 [119]	GCST011370

There are several limitations in this gene-to-phenotype approach, as the phenotypes of neuropsychiatric disorders are often heterogeneous and influenced by multiple factors, e.g., environmental conditions and developmental stages. In general, GWAS studies identify loci associated with traits but often fail to address causation [22, 23], i.e., the majority of SNPs identified for *GPR139* are located in intronic or noncoding regions. This is a bottleneck in most GWAS for determining the functional relevance of SNPs, although incorporating high-throughput in vitro and in vivo models and the utilization of massively parallel reporter assays and sequencing techniques may address this limitation [24]. In the context of neuropsychiatric disorders, GWAS are further limited by the risk of bias from self-reporting and a lack of gold standards in the classification of symptomatology, as well as a lack of diversity in study populations and limitations in cohort size. Nevertheless, GPR139 remains a target of interest for understanding the pathophysiology of these neuropsychiatric disorders, and there is an urgent need for a comprehensive review of the current findings associated with GPR139.

### Ligand Binding Activity of GPR139

**Fig. 1:**
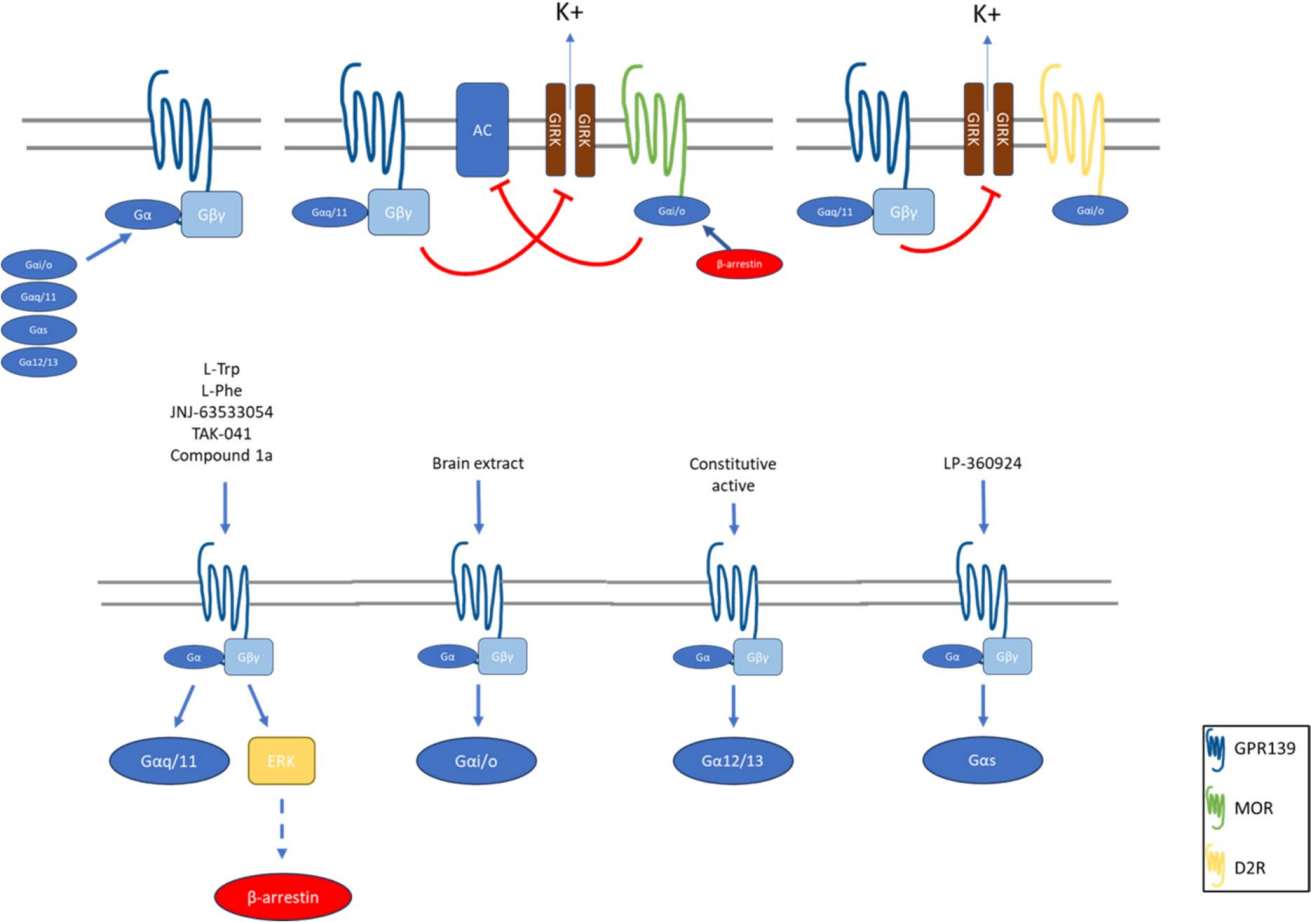
GPR139 interactions and signaling pathways. Top left: GPR139 exhibits G-protein promiscuity with G_i/o_, G_q/11_, G_s_, and G_12/13_. Top middle and right: GPR139 cross-talk with μ-opioid receptor (MOR) and dopamine D 2 receptor (D2R) via the inhibition of the downstream effectors adenylyl cyclase (AC) and G protein-coupled inwardly rectifying potassium channel (GIRK), which results in the negative regulation of MOR and D2R. Bottom: GPR139 agonists activate different signaling pathways. These agonists were tested in different cell lines; the results of which are summarized in Table 2

Multiple studies have investigated GPR139 signaling and function using pharmacological assays and virtual screening. These studies have identified reference agonists and characterized their potency in various in vitro assays (see Table 2 and Fig. 1), including Ca^2+^ mobilization and inositol monophosphate accumulation [25].

**Table 2:** Summary of characterized probable endogenous and surrogate ligands for GPR139 and their EC50 values from Ca^2+^ mobilization assays

Ligands	Models	EC50 (µM)	Citations
**Endogenous ligands**			
**Amino acids**			
l-Trp	Pharmacophore	220	Isberg et al., 2014 [26]; Liu et al., 2015 [6]
l-Phe		320	
**Surrogate agonists**			
TAK-041	CHO-T-Rex cell line	0.022	Reichard et al., 2021 [31]
JNJ-63533054	Human HEK293F cell line	0.016	Dvorak et al., 2015 [5]; Liu et al., 2015 [6]
	CHO-T-Rex cell line	0.013	Pallareti et al., 2023 [25]
LP-360924	Human HEK293F cell line	> 10	Hu et al., 2009 [36]
Compound 1a	CHO-K1 cell line	0.039	Shi et al., 2011 [33]; Shehata et al. 2016 [34]; Bayer-Andersen et al., 2016 [35]
	CHO-T-Rex cell line	0.021	Pallareti et al. 2023 [25]
Compound 2	CHO-K1 cell line	0.53	Bayer-Andersen et al., 2016 [35]
Compound 3	CHO-K1 cell line	0.85	Bayer-Andersen et al., 2016 [35]
AC4	CHO-K1 cell line	0.22	Nohr et al., 2017 [27]
	CHO-T-Rex cell line	0.192	Pallareti et al. 2023 [25]
DL43	CHO-K1 cell line	0.36	Shehata et al. 2016 [34]
	CHO-T-Rex cell line	0.264	Pallareti et al. 2023 [25]
**Surrogate antagonists**			
LP-471756	CHO-K1 cell line	0.64	Hu et al., 2009 [36]
	CHO-T-Rex cell line	2.933	Pallareti et al. 2023 [25]
LP-114958		0.67	
NCRW0001-C02	CHO-K1 cell line	0.42	Wang et al., 2015 [37]
	CHO-T-Rex cell line	0.513	Pallareti et al. 2023 [25]
NCRW0005-F05	CHO-K1 cell line	0.21	Wang et al., 2015 [37]
NCRW0008-C04		2.1	
NCRW0095-F03		0.83	
NCRW0105-E06		0.43	
	CHO-T-Rex cell line	0.512	Pallareti et al. 2023 [25]
Compound 4	CHO-K1 cell line	7.4	Bayer-Andersen et al., 2016 [35]
JNJ-3792165	Human HEK293F cell line	0.13	Nepomuceno et al., 2018 [30]
	CHO-K1 cell line	0.025	Pallareti et al., 2023 [25]

GPR139 was shown to be activated by the endogenous ligands l-tryptophan (l-Trp) and l-phenylalanine (l-Phe) [6, 7, 26, 27]. Isberg et al. reported EC50 values of 220 and 320 µM for l-Trp and l-Phe, respectively [26], whereas Liu et al. reported EC50 values within the range of 30–300 µM for l-Trp and l-Phe. Liu et al. hypothesized that GPR139 plays a regulatory role in detecting intrinsic concentrations of l-Trp and l-Phe in the brain [6]; however, further empirical studies are needed to draw any significant conclusions. Importantly, although l-Trp and l-Phe are metabolite precursors of serotonin and dopamine, there is currently no evidence that GPR139 is activated by both monoaminergic neurotransmitters. Luciferase reporter activity revealed that GPR139 has a greater preference for G_q/11_ coupling than for Gs coupling [4]. Stoveken et al. reported that GPR139 activates both the G_q/11_ and G_i/o_ families of G proteins [2]. A synthetic agonist, JNJ-63533054, has been validated in several studies using different cell lines, i.e., HEK293F and CHO-K1, with EC50 values of 0.016 and 0.013, respectively [5, 6, 25]. An in silico analysis by Zhou et al. reported a peculiarity in the ligand binding of GPR139, where JNJ-63533054 [5] was found to interact with GPR139 in different conformations, i.e., JNJ-63533054 has only one binding pose where the receptor is coupled with Gs/q, whereas the compound presents two diverse binding poses when the receptor is coupled with Gi as follows: (i) similar to the conformation when coupled with Gs/q and (ii) a flipped amide bond that results in a change in the compound conformation [28]. Interestingly, JNJ-63533054 binds to GPR139 at the same site as the endogenous ligands l-Trp and l-Phe do [29]. GPR139 was also previously reported to be activated by the HFRW peptides: adrenocorticotropic hormone (ACTH), α-melanocyte-stimulating hormone (α-MSH), and β-MSH [27]; however, a Ca^2+^ mobilization study by Nepomuceno et al. demonstrated that GPR139 is not activated by these peptides at physiologically relevant concentrations [30].

Molecular docking analyses revealed the same binding site and similar molecular dynamics for TAK-041 (also known as Zelatriazin or NBI-1065846, EC50 = 0.022) [31], a compound used for a phase I clinical trial for adult schizophrenia, i.e., the oral administration of TAK-041 significantly improved anxiety and depressive symptoms in schizophrenic patients, and minimal treatment-associated adverse reactions (headache and drowsiness) were been reported [32]. However, patients showed no significant improvement in cognitive function following TAK-041 administration [32]. TAK-041 was also used in a phase II clinical trial for anhedonia in MDD patients; however, the compound failed to meet the primary endpoint. In addition to JNJ-63533054 and TAK-041, several other surrogate agonists and antagonists have been developed and validated in several in vitro studies [30, 33–37]; however, the downstream signaling pathways of these compounds remain uncharacterized, and their physiological effects have yet to be validated in animal models. Although ligand‒receptor binding studies often provide useful pharmacological insights into the compounds tested, it is important to note that in vitro assays often fail to provide physiological relevance, i.e., the saturation of target receptors, type of cell line used, high ligand concentrations, and other experimental conditions may not accurately represent realistic physiological conditions in native tissue compared with in vivo tests.

### GPR139’s Interactions with Other Receptors

GPCRs can exercise modulatory functions toward other receptors by coexpressing in close proximity or by forming receptor complexes, i.e., dimerization or polymerization, either homogenously or heterogeneously, thus increasing the diversity of receptor‒receptor interactions, novel receptor pharmacology, and subsequent downstream signaling pathways [38, 39]. Like many other GPCRs, GPR139 is likely to exhibit promiscuity with multiple G proteins, such as G_q_, G_s_, and G_i_. Currently, the only known interactions of GPR139 are with the μ-opioid receptor (MOR) and dopamine receptor 2 (D2R), which are summarized in Fig. 1.

In mice, the coexpression of GPR139 with MOR has been observed, and GPR139 was found to inhibit MOR signaling cascades [40]. Wang et al. reported the expression of a GPR139 ortholog in the nematode *Caenorhabditis elegans* as a conserved inhibitor of MOR activity [40]. In this study, GPR139 expression was shown to have a direct effect on morphine-induced membrane hyperpolarization, where hyperpolarization was reduced when GPR139 expression was equivalent to that of MOR and virtually obliterated hyperpolarization when GPR139 was overexpressed [40]. However, this study reported that the expression of GPR139 had no effect on MOR localization to the cell membrane and suggested that GPR139 inhibits MOR signaling via the recruitment of β-arrestin and the modulation of G-protein activation [40]. Similarly, Stoveken et al. reported that GPR139 inhibits MOR signaling via the G_q/11_ pathway by modulating common downstream effectors such as adenylyl cyclase (AC) and G protein-coupled inwardly rectifying potassium channels (GIRKs) [2]. Previously, the G_q/11_ and G_i/o_ pathways were established for ion channels and AC, where interactions between G_i/o_-coupled receptors and G_q/11_-coupled receptors result in G_q/11_ signaling [41, 42]. As such, it is logical to hypothesize that GPR139 coupled with G_q/11_ may interact with other G_i/o_-coupled receptors in proximity, such as D2R.

The coexpression of GPR139 and D2R across multiple brain regions was previously established in an RNA-Seq study [9]. Dao et al. reported the inhibition of D2R signaling by GPR139, where HEK293T cells coexpressing GPR139 and D2R did not respond to dopamine stimulation and GPR139 expression did not influence D2R saturation in the membrane bilayer [43]. This finding revealed that GPR139 coupled with G_q/11_ overwrites signaling by D2R coupled with G_i/o_ at GIRK. Wang et al. reported an increase in intracellular Ca^2+^ levels in response to JNJ-63533054 in GPR139-transfected D2R-expressing HEK293 cells [9]. Interestingly, quinpirole, a D2R agonist, had no effect on cells transfected with D2R alone; however, it elicited an increase in Ca^2+^ when D2R cells were cotransfected with G_qi5_ (EC50 = 1.72 ± 1.11 nM). Quinpirole was able to elicit an increase in Ca^2+^ in cells that coexpressed GPR139 and D2R in the absence of G_qi5_ (EC50 = 2.04 ± 1.32 nM), suggesting that GPR139 is able to modulate D2R signaling by recruiting G_q_ to D2R. However, the physiological effects of GPR139 and D2R coexpression have not been examined in animal models. Importantly, GPCR expression varies across different cell lines and has a direct effect on the potency of agonists [44].

Although the coexpression and interaction of GPR139 with MOR and D2R has been empirically reported, it is uncertain whether GPR139 forms receptor dimers with MOR and D2R. Susens et al. reported the exclusive presence of GPR139 homodimers in CHO-K1 cells, which are present as monomers in HEK-293 cells [7]. This finding suggests that different cell types may affect receptor cross-talk and interactions, as effector systems may vary across cell populations owing to various factors, including receptor compartmentalization and the cellular microenvironment. Similar to ligand‒receptor binding assays, the study of receptor cross-talk and interaction often requires artificial modulation of receptor saturation in a model with limited throughput and low reproducibility. Additionally, confirming the configuration of receptor complexes by molecular techniques has been challenging, as conformational transitions often occur at the µs timescale [45], thus suggesting the need to utilize large-scale molecular dynamics simulations or electron microscopy to empirically validate the implications of GPR139–MOR or GPR139–D2R heteroreceptor complexes.

### GPR139 in Animal Studies

In primates and nonprimate mammals, GPR139 expression is unique to the thalamic region, where it is highly expressed. Transcriptomic profiling of the mouse cortex revealed that GPR139 protein expression is silent across cortical regions [46], suggesting a phylogenetically ancient role that is central to thalamic functions. In the mouse habenula, *Gpr139* mRNA expression follows a mediolateral gradient, where it is concentrated in the medial habenula (mHb) and dispersed in the lateral habenula (lHb) [6]. Interestingly, this expression pattern has been observed in nonmammalian animals, where *gpr139* mRNA expression follows the ventral habenula (vHb) in zebrafish [10]. Transcriptomic profiling and behavioral studies have shown that mHb is homologous to the nonmammalian dorsal habenula (dHb) and that lHb is homologous to the VHb [47, 48]. In a mixed-methods study, similar tissue distributions of *GPR139/Gpr139* and *D2R/D2r* mRNA were reported in several thalamic brain regions in humans and rodents via RNA sequencing, although *D2R* expression is significantly greater across tissues in both humans and rodents [9]. Interestingly, GPR139 and D2R coexpression at the cellular level has been reported in several nonthalamic brain regions, including the VTA, olfactory tubercle, substantia nigra, and hippocampus, in mouse tissue via in situ hybridization [9]. Table 3 summarizes the regions with the highest *GPR139/Gpr139/gpr139* mRNA expression in humans, rodents, mice, and zebrafish. Nevertheless, these findings raise two concerns: (i) GPR139-expressing cell types remain unidentified, and (ii) the anatomical characterization of *GPR139/Gpr139/gpr139* mRNA remains understudied. Although transcriptomic profiling provides significant insights into the unique expression pattern of *Gpr139*, there is a need to study the physiological role of GPR139 using in vivo models to better understand its gene‒phenotype correlations.

**Table 3:** Summary of the brain regions with the highest reported expression of GPR139/*gpr139* and the type of assay used

Brain region (alphabetical order)	Human	Rodent	Mice	Zebrafish
Brainstem	RNA-Seq [9]	-	RNA-Seq [9]	-
Habenula (lateral/ventral ortholog)	-	-	-	ISH [8, 10]
Habenula (medial/dorsal ortholog)	-	ISH [5, 8]	ISH [4]; Northern blot [7]	-
Hippocampus (CA1)	-	ISH [5]	-	-
Hippocampus (CA3)	-	ISH [5]	-	-
Hippocampus (dentate gyrus)	-	ISH [5]	-	-
Hypothalamus	qPCR [4, 6]; RNA-Seq [9]	qPCR [6]; RNA-Seq [9]	RNA-Seq [9]	-
Insular cortex	qPCR [6]	-	-	-
Medulla	Northern blot [7]; RNA-Seq [9]	-	RNA-Seq [9]	-
Olfactory	RNA-Seq [9]	-	RNA-Seq [9]	-
Pituitary	qPCR [4, 6]	qPCR [6]; RNA-Seq [9]	-	-
Spinal cord	RNA-Seq [9]	-	-	-
Septum (medial)	-	-	qPCR, IHC, Western blot [55]	-
Striatum	qPCR [4, 6]; RNA-Seq [9]	qPCR [6]; ISH [5]; RNA-Seq [9]	ISH [4]; RNA-Seq [9]	-
Substantia nigra	RNA-Seq [9]	qPCR [6]	-	-
Thalamus	qPCR [6]; RNA-Seq [9]	qPCR [6]; RNA-Seq [9]	RNA-Seq [9]	-
Zona incerta	-	-	ISH [4]	-

*IHC* immunohistochemistry, *ISH* in situ hybridization, *qPCR* quantitative-PCR, *RNA-Seq* RNA-sequencing

In knockout studies, Dao et al. reported that *Gpr139*^−/−^ mice presented increased schizophrenia-like symptoms, including hyperactivity, anxiety-like behavior, and social interaction deficits, and the administration of the D2R antagonist haloperidol rescued these behavioral deficits [43]. Atienza et al. reported behavioral deficits in *Gpr139*^−/−^ mice, especially in the paradigms of motivation and self-neglect [49]. *Gpr139*^−/−^ mice also exhibited altered rapid eye movement (REM) sleep under normal conditions, where significantly fewer but longer REM episodes were observed, and showed a minimal response in sleep suppression induced by selective serotonin reuptake inhibitors (SSRIs) [50], suggesting that GPR139 is a potential target of treatment-resistant depression and schizophrenia, as well as other neuropsychiatric disorders.

Münster et al. reported that TAK-041 increased reward-seeking behavior in starved mice, as well as in mice that exhibited learned helplessness induced by chronic social stress [51]. TAK-041 has also been reported to rescue anhedonia-like behavior and social interaction deficits in rodent models of schizophrenia. TAK-041 has also been reported to reduce amphetamine-induced dopamine concentrations in the synaptic cleft; however, there are no data available on its effects on endogenous dopamine mechanisms, e.g., synthesis, vesicle packing, and trafficking. There are currently no reports on the effectiveness of TAK-041 toward the serotonergic system. The role of serotonin and dopamine in reward-seeking behavior has been more commonly studied [52], especially in the context of the habenula and its anatomical connection with the serotonergic raphe nuclei and dopaminergic centers, i.e., the substantia nigra pars compacta (SNc) and VTA.

JNJ-63533054 was found to reduce the latency of nonrapid eye movement (NREM) and increase the duration of NREM sleep without affecting REM sleep in rodents [53]. JNJ-63533054 reportedly rescued addiction-like behaviors in alcohol-dependent rats, suggesting that GPR139 may be a potential therapeutic target for alcohol use disorder. Interestingly, JNJ-63533054-treated rats showed a reduction in interest in the urine sniffing test, as well as anxiolytic-like effects in the marble burying test [54]. This phenomenon bears similarities to anhedonia phenotypes; nevertheless, further behavioral validation is needed. A recent study by Mu et al. reported the rescue of cognitive deficits in an Alzheimer’s disease (AD) mouse model injected with Aβ_1–42_ aggregates via intraventricular administration of JNJ-63533054, as well as the amelioration of neuronal apoptosis and synaptotoxicity of medial septum cholinergic neurons in APP/PS1 transgenic mice via the overexpression of the GPR139 receptor [55]. This study revealed that GPR139 is a promising treatment target for neurodegenerative diseases; however, the effects of JNJ-63533054 in human subjects have not been validated through clinical trials.

JNJ-63533054 administration was also tested in a nonmammalian animal model, where the agonist reportedly attenuated alarm-substance-induced avoidance behavior and induced spatial nonpreference without significantly affecting locomotion in zebrafish [10], similar to the rodent model of learned helplessness conducted by Münster et al. [51]. Interestingly, the coadministration of JNJ-63533054 with the synthetic antagonist NCRW0005-F05 interfered with fear conditioning in adult zebrafish by reducing locomotion and increasing calcium transient peak amplitudes in acute habenula slices postadministration of either JNJ-63533054 or NCRW0005-F05 [56]. Roy et al. reported that NCRW0005-F05 administration significantly ameliorated alarm-substance-induced avoidance; however, the physiological effects of the antagonist have yet to be validated in a mammalian model [56]. A microdialysis study of the mouse cortex and nucleus accumbens after acute administration of JNJ-63533054 revealed no effect on endogenous dopamine or serotonin release [54]; however, further validation of chronic administration is needed to elucidate the effects of JNJ-63533054.

To date, the behaviors of *Gpr139* knockout model animals have been shown to be similar to those of wild-type animals treated with either TAK-041 or JNJ-63533054; however, further validation is needed to confirm the pharmacological correlation between the ligands and their receptors. Future studies should also consider ligand‒receptor conservation across different species, as the aforementioned surrogate ligands are designed on the basis of human GPR139. Nevertheless, the physiological function of GPR139 remains elusive.

### GPR139 in an Evolutionary Context

GPR139 ortholog expression has been reported in several invertebrates, including the nematode *C. elegans* [40] and cnidarian hydra [57]. Receptor clustering analyses revealed GPR139 ortholog expression in several protostomes, e.g., amphioxi, *Branchiostoma floridae* [58], and ambulacrarians [59]. These GPCR orthologs were found to be evolutionarily related to the receptors of the aromatic neuropeptides allatostatin-B (AST-B) and proctolin, which are unique to protostomes. AST-B, also known as myoinhibitory peptide (MIP), is an aromatic neuropeptide commonly investigated in arthropods and mollusks, where it acts as a ligand for the sex peptide receptor (SPR) to regulate arousal and sleep [60]. On the other hand, proctolin is an excitatory aromatic neuropeptide that increases muscle contraction in arthropods and modulates neuron firing patterns in peripheral tissue [61]. GPR139 has also been considered orthologous to FMRFamide receptors (herewith referred to as FMRFa-Rs) in invertebrates via phylogenetic clustering studies [62]. FMRFa-Rs are common in all nonvertebrate animals and are associated with a range of innate primitive functions, e.g., reproductive function, sleep, and food-seeking behavior [63]. This enhances the theory of alert consciousness associated with receptor selectivity toward aromatic ligands [64]. Therefore, it is not illogical to suggest that GPR139 similarly displays selectivity toward aromatic ligands and has a physiological role in regulating similar cognitive functions, e.g., attention, alertness, and reward. As mentioned previously, we hypothesize that GPR139 is a chemoreceptor that is sensitive to changes in the levels of endogenous monoaminergic or trace-amine systems within the local microenvironment. Importantly, future studies should consider the differences in GPR139 functionality in relation to ligand availability, expression of cell types, and stage of development of animal models.

## Discussion

Neuropsychiatric disorders such as depression, schizophrenia, and ADHD share a range of emotional and behavioral symptoms, including a significant lack of self-esteem and motivation, anhedonia, loss of appetite, social withdrawal, sleep disturbances, and more. The overlap of symptoms across these neuropsychiatric disorders is often poorly characterized, inconsistent across treatment approaches, and easily mistaken for each other. For example, anhedonia-like behavior is common in both depressive and schizophrenic patients; however, there is no clear consensus on the characterization of anhedonistic traits in either group [65–67]. According to Castle and Bosanac, depressive patients are able to define the shift in interests and sometimes exhibit rumination tendencies or emotional changes associated with their loss of interest, whereas schizophrenic patients describe their interests without motivational affect and exhibit a platonic emotional state, which, in some cases, develops into apathy in the long run [68]. Insomnia and sleep disturbances are other common traits found in depressive and schizophrenic patients, where sleep markers are found to be consistent in both neuropsychiatric disorders: alterations in NREM sleep, decreases in REM latency, increased REM duration, etc. [69–72]. Other traits, such as lateral ventricle volume changes, alcohol misuse disorder, and inattention, are considered transdiagnostic markers of these neuropsychiatric disorders; however, there is a lack of consensus on the characterization of these markers for each of these neuropsychiatric disorders. Thus, despite intensive research and clinical trials over the past seven decades, the pathophysiology of these disorders has remained elusive and highly heterogeneous, compounded by their high risk of comorbidity [73, 74].

### Habenula

Derived from the Latin *habena* (meaning little rein) owing to its elongated shape, the habenula is an evolutionarily conserved, asymmetrically paired cluster of nuclei located in the epithalamic region of vertebrates [75]. The habenula has been a region of interest across various experimental and clinical studies of symptomatology associated with several neuropsychiatric disorders, e.g., depression, schizophrenia, and autism spectrum disorders [76–78]. The habenula has been associated with a wide range of behavioral phenotypes that can be broadly classified into (i) cognitive phenotypes, e.g., fear learning, reward prediction, and motivation [10, 79–81]; (ii) social phenotypes, e.g., social interaction and avoidance, aggression, and hierarchical organization [82–84]; (iii) metabolic phenotypes, e.g., feeding and addiction [47, 85–87]; (iv) circadian rhythmicity, sleep, and states of consciousness [88–91]; and (v) reproductive behaviors [47]. The main efferent output of both habenulae forms a dense white matter tract known as the fasciculus retroflexus (FR), also known as the habenula-interpeduncular tract. The neuronal populations of mHb and lHb can be distinguished on the basis of cytoarchitectural, neurochemical, and transcriptomic characteristics [92–94]. mHb neurons receive inputs from the septal nuclei and project to the interpeduncular nucleus (IPN), whereas lHb neurons connect several forebrain and hindbrain structures to the monoaminergic systems of serotonin and dopamine, i.e., the raphe nuclei, VTA, and SNc.

Western blot analyses of mouse embryonic tissue reveal a distinct expression pattern in which GPR139 expression is most prominent in the central nervous system between stages E11 and 15 and then decreases by E17 [7]. During mouse embryonic development, E11–15 corresponds to the time frame in which the habenula undergoes differentiation (E11.5–13.5) and the fasciculation of habenula axons to the SNc terminal by E15.5 [95]. Previous studies reported axonal degeneration and compromised fascicular integrity of habenula afferents in rodents following self-administration or chronic exposure to amphetamine, cocaine, and nicotine [96–99]. Lesions of the FR result in increased incentive salience, which mimics withdrawal from chronic administration of abuse substances [100]. Importantly, lesion studies have produced heterogeneous outcomes in similar animal models. Thus, contextual analyses at the habenula and FR levels, e.g., profiling of habenula and glial populations, are crucial to generate a ground-truth understanding of animal behavior toward opiates and other nonopiate substances [101]. This can be addressed via the use of a suitable animal model, e.g., zebrafish, where live imaging of cell populations in the ms timeframe can be conducted while simultaneously quantifying animal behavior. Nonetheless, it is logical to hypothesize that GPR139 might have physiological relevance in the habenula and FR, particularly in terms of fascicular integrity and sensitivity to opiates and other substances of abuse.

### Potential Physiological Roles of GPR139

#### Regulation of Addiction and Withdrawal

*GPR139* risk variants (rs72771074 and rs2764771) have been found to be associated with alcohol use disorder in two independent populations [18, 19], thus demonstrating that GPR139 has a physiological role in the neurocircuitries of reward and addiction. Evidence of the GPR139–MOR interaction provides crucial insight into the physiological role of GPR139 [40]. GPR139 interaction and coexpression with MORs have been previously reported, where GPR139 inhibits MOR signaling and overexpression of GPR139 results in an impediment to MOR trafficking [40, 102]. Dao et al. reported that *Gpr139*^−/−^ mice present a reduction in opioid withdrawal symptoms, and the MOR antagonist naltrexone rescued all behavioral deficits in *Gpr139*^−/−^ mice, further confirming the negative regulatory effect of GPR139 on MOR activity [43]. MORs are key regulators in the opioid system and interact with the endocannabinoid system [103]. In addition to being commonly associated with circuitries of reward and nociception, MORs have also been implicated in metabolism, alcohol use, and dependence, where MORs mediate the enhancement of the pleasurable effects of nonopioid drugs of abuse, including alcohol, cannabinoids, and nicotine [104–107]. MORs are highly expressed in the FR, and their expression is ubiquitous throughout mHb neurons [108]. Optogenetic stimulation of MOR-positive neurons in the mHb of rodents triggers aversive avoidance and despair-like responses with no anxiety-related effects, and stimulation at the dorsal raphe nucleus terminal of MOR-positive mHb neurons increases anxiety levels without affecting other behaviors, revealing the involvement of two separate pathways in aversive learning and its affective behavioral phenotypes [109]. To gain further insight into the role of GPR139 and its effect on MOR signaling, it is imperative for future optogenetic studies of MOR-positive neurons to consider behavioral changes in GPR139 gene knockout models. GPR139 agonism may act as a preventive therapeutic avenue for regulating withdrawal syndrome and addiction symptoms through its ability to inhibit MORs via the activation of G_q/11_ and the recruitment of β-arrestin to MORs.

In the habenula, cholinergic neurons corelease glutamate and acetylcholine to downstream neurons [110], and the habenula plays an inhibitory role in the VTA and nucleus accumbens (NAc) populations [111, 112]. Zapata et al. reported that the activation of Hb cholinergic neurons ameliorates impulsive self-administration of cocaine [113]. D2R expression in the NAc cholinergic neurons of mice susceptible to cocaine-seeking behavior is significantly greater than that in their resilient counterparts [114]. On the basis of the findings of previous studies, we hypothesize that GPR139 agonism and cross-talk with D2Rs play a neuroprotective role against neuronal damage, apoptosis, and synaptoxicity upon exposure to substances of abuse and other neurotoxins. Here, we hypothesize that the differential activation of GPR139 in Hb neuronal populations may modulate dopamine release at downstream targets and increase resilience toward impulsive reward-seeking behavior and other withdrawal symptoms.

#### Regulation of the States of Consciousness and Sleep

Preliminary evidence from Wang et al. revealed that the synthetic GPR139 agonist JNJ-63533054 facilitates the initiation of the sleep cycle [50]. Interestingly, there are varying effects of the agonist on NREM sleep depending on the time of agonist administration, where significant reductions in NREM latency and duration are only observed when JNJ-63533054 is administered during the day (light cycle) and there are no effects on NREM latency and duration when JNJ-63533054 is administered at the onset of the dark cycle [50]. Currently, there are no data on the effects of TAK-041 on sleep patterns. REM sleep is significantly reduced in *Gpr139*^−/−^ mice compared with their wildtype conspecifics, and their NREM sleep is not altered [50]. Interestingly, previous FR lesion studies reported similar outcomes, i.e., FR lesions fragmented REM sleep bouts and reduced REM sleep by 79% but had no significant effect on NREM sleep [115, 116]. These findings suggest that GPR139 expression corresponds to the physiological role of the FR. *Gpr139*^−/−^ mice also demonstrate reduced sensitivity to citalopram- or fluoxetine-induced REM sleep suppression and increased sensitivity to amphetamine, which is a stimulant that promotes alertness [50]. From a phylogenetic perspective, GPR139 is orthologous to the AST-B and FMRFamide receptors, both of which are regulators of sleep and arousal [59, 62]. These findings suggest that GPR139 expression and signaling in the habenula and FR may be regulated by the circadian rhythm. Yamamoto suggested a hypothesis in which GPR139 detects l-Trp level changes in the brain, which subsequently contributes to central fatigue and reduced alertness, which is considered a common phenotype in patients with neuropsychiatric disorders [117]. On the basis of current evidence that GPR139 activity regulates opioid withdrawal effects and nociception, the selectivity of GPR139 toward aromatic ligands and its interactions with other receptors may modulate dynamic changes across states of consciousness. Nonetheless, the role of GPR139 in regulating sleep should be further examined in knockout models before drawing decisive conclusions.

#### Neuroprotective Role Against Neurotoxins and Protein Aggregates

The role of GPR139 in neurodegenerative diseases such as Parkinson’s disease (PD) and AD has been examined using different experimental models. Bayer Andersen et al. demonstrated that GPR139 plays a neuroprotective role in an 1-methyl-4-phenylpyridinium [MPP (+)] -induced neurotoxicity model of PD, where acute slice midbrain dopaminergic neuronal cultures pretreated with a GPR139 agonist significantly withstood the apoptosis induced by 1 µM MPP (+) as the agonist dosage increased, and the coadministration of 10 µM of a GPR139 antagonist with MPP (+) significantly decreased cell viability [35]. However, these findings have yet to be validated using other agonists, such as TAK-041 and JNJ-63533054, and the experimental conditions have yet to be reproduced in a different cell line. A recent study by Mu et al. reported similar neuroprotective effects of the agonist JNJ-63533054 in the medial septum cholinergic neurons of an AD murine model, where JNJ-63533054 rescued Aβ_1–42_-induced neurotoxicity and apoptosis, and the overexpression of GPR139 in this cholinergic population rescued cognitive impairment and neuronal damage [55]. Findings from these two independent studies suggest that GPR139 plays a neuroprotective role in neurodegenerative diseases and that its use as a preventive treatment against neuronal damage is due to its selective binding to GPR139. Modulating GPR139 activity could influence D2R signaling, suggesting a novel approach to managing motor symptoms or addressing the nonmotor symptoms associated with PD, such as mood disturbances or impulse control disorders. Nevertheless, further experimental validation in other models is needed to draw any significant conclusions on the physiological effects of GPR139 in these neuronal populations.

## Conclusions

GPR139 is a relatively understudied GPCR that is specifically expressed within the diencephalon and is activated by the amino acids l-Trp and l-Phe. However, like many other GPCRs, the physiological role of GPR139 in the pathophysiology of neuropsychiatric and behavioral disorders remains relatively elusive, as current studies are considered preliminary and not currently validated in humans or other vertebrate phylogenetic taxa. GPR139 has been found to modulate MOR signaling and withdrawal symptoms in rodents in response to opiates and alcohol. While direct evidence linking the chronic administration of opiates and other nonopioid substances to fascicular degeneration in rodents is lacking, it would be interesting to validate this finding in *Gpr139*^−/−^ animal models. This area of research remains highly speculative and would benefit from further empirical investigation to establish a physiological role for GPR139. It is therefore crucial for future studies to consider the multifaceted aspects of receptor pharmacology and physiology to allow a greater comprehension of GPR139 and its implications in health and disease.

## Data Availability

No datasets were generated or analysed during the current study.
